# The Relevance of Life Cycle Assessment Tools in the Development of Emerging Decarbonization Technologies

**DOI:** 10.1021/jacsau.3c00276

**Published:** 2023-09-28

**Authors:** Javier Fernández-González, Marta Rumayor, Antonio Domínguez-Ramos, Angel Irabien, Inmaculada Ortiz

**Affiliations:** Department of Chemical and Biomolecular Engineering, Universidad de Cantabria, Avenida Los Castros s/n, 39005 Santander, Spain

**Keywords:** life cycle assessment, planetary boundaries, CO_2_ recycling, decarbonization, emerging technologies

## Abstract

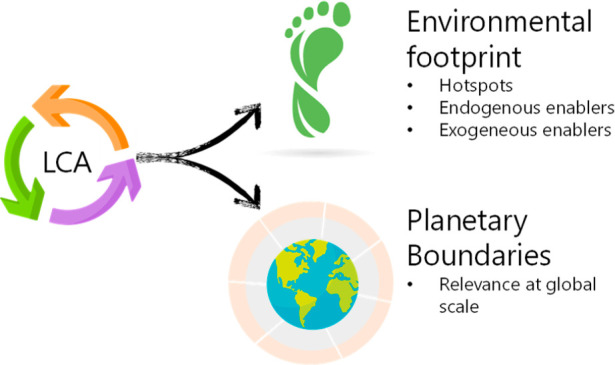

The development of emerging decarbonization technologies requires advanced tools for decision-making that incorporate the environmental perspective from the early design. Today, Life Cycle Assessment (LCA) is the preferred tool to promote sustainability in the technology development, identifying environmental challenges and opportunities and defining the final implementation pathways. So far, most environmental studies related to decarbonization emerging solutions are still limited to midpoint metrics, mainly the carbon footprint, with global sustainability implications being relatively unexplored. In this sense, the Planetary Boundaries (PBs) have been recently proposed to identify the distance to the ideal reference state. Hence, PB-LCA methodology can be currently applied to transform the resource use and emissions to changes in the values of PB control variables. This study shows a complete picture of the LCA’s role in developing emerging technologies. For this purpose, a case study based on the electrochemical conversion of CO_2_ to formic acid is used to show the possibilities of LCA approaches highlighting the potential pitfalls when going beyond greenhouse gas emission reduction and obtaining the absolute sustainability level in terms of four PBs.

## 1 Introduction

Recent global environmental changes suggest that the Earth is passing into a new geological time, the Anthropocene, where humans constitute the dominant driver of change to the Earth’s system.^1^ Among global pressures, the Earth’s climate is an existential threat to Europe and the entire world. Emerging decarbonization technologies, especially CO_2_ conversion technologies, will play a crucial role in transitioning to a resilient planet. According to the recent IPCC scenarios,^2^ keeping global warming below 1.5 °C requires net-zero anthropogenic greenhouse gas (GHGs) emissions by around 2050. Hence, deploying decarbonization technologies will require innovation beyond those current commercial technologies (e.g., renewable electricity, electrolytic hydrogen production, ...).^3^ Pathways to achieve a net-zero economy are countless, but not all are optimal for operating sustainably and are associated with higher risks and uncertainties.^4^ In this sense, technology developers have to make several choices throughout technology scaling-up processes which can further impact the economic and environmental performances during the implementation stage. Hence, the delivery of sustainable low-carbon technologies requires the development of both new technologies and transparent methods to estimate emission reductions and targets in different scenarios. Furthermore, in order to overcome new challenges, the benefits of technologies should contemplate global sustainability ensuring the full resilience of the Earth.

Despite the fact that several environmental metrics and frameworks have been applied over the last few decades,^5,6^ the prospective Life Cycle Assessment (LCA) is currently the reference tool to assess the environmental impacts as it considers the full life cycle avoiding shifting burdens.^7^ Since the normalization through the ISO standards of LCA,^8,9^ the tool use has strengthened and become widespread, and several applications and approaches have evolved in recent years.^10^ Prospective LCA is currently applied to evaluate the environmental benefits early on the development of emerging technologies besides its traditional use as a measurement tool to determine the environmental impacts of products and services.^10,11^ Recent LCA developments in the literature have moved toward new approaches, such as the elucidation of a better environmental performance stage of specific technologies,^12,13^ the selection of sustainable pathways among a number of options,^14,15^ or even identifying hotspots and targets unlocking the full potential of the incumbent technology in hard-to-abate sectors.^16,17^ However, the main challenge of any prospective LCA is dealing with uncertainty since emerging technologies have not been tested in real operating environments.^18−20^ The hotspots identified by LCA are the key areas of interventions to be considered for reducing impacts and establishing target values of key performance indicators. However, most of the prospective LCA studies are based on a limited set of midpoint categories, so they do not provide the distance to the ideal reference sustainable state.

One aspect that is gaining momentum within the LCA community is the possibility of calculating absolute sustainability metrics. The concept of Planetary Boundaries (PBs) has now arisen in the LCA framework to identify the distance to the ideal reference state. The PB framework was developed in 2009 by Rockström and colleagues^21^ and improved in 2015.^22^ By definition, the PBs determine whether the levels of a set of anthropogenic perturbations remain below the risk of destabilization of the Earth system, including land, oceans, atmosphere, and life. The set of ecological thresholds that include 11 Earth system processes may be used to quantify critical impacts for the resilience of the planet. Hence, impacts at a global scale through PBs can be used for identifying safe whole targets in LCA contexts, which means assessing the potential of interventions to ensure sustainability. The recent combined approach of LCA-PB may answer whether a technology is truly sustainable in absolute terms. The current trend tries to include the assessment of the absolute sustainability level of decarbonization pathways for the chemical industry, which has been appointed as a key strategy to guide development as well as policy-making.^23^ Several studies that apply the PB framework for decision-making have been published in recent years.^24−28^ Galán-Martin et al. applied PB-LCA to evaluate the transition of the petrochemical industry to renewable carbon-based, highlighting the opportunities to incorporate PBs in the decision-making when assessing large-scale decarbonization routes. D’Angelo et al. assessed the absolute sustainability performance of low-carbon ammonia synthesis routes from the PB-LCA perspective. Earth impacts caused by the large amount of metals that are required by low-carbon technologies have also been evaluated by Schenker et al. under a PB framework, identifying challenges related to metal in the PB dimension. Engström et al. used the PB framework to analyze the carbon pricing impact on the Earth system beyond its effects on just carbon emissions and found that carbon pricing may alleviate other planetary threats. Bachmann et al. evaluated circular strategies within the plastic sector, such as recycling, biomass utilization, and CO_2_ utilization, defining a pathway that can lead to a safe operating space in 2030. Since the chemical sector has to be shifted toward more sustainable technologies, special attention may be paid to developing those that close the anthropogenic loop under the circular economy principles.

This study is focused on the demonstration of the potential of the LCA tool to elucidate developing scenarios of sustainable processes and services. We focus on the main LCA perspectives going from its prospective approach to the recent link with the planet limits. The study will focus on the LCA tool from the traditional approach to the recent LCA-PB perspective. This overview is applied to the CO_2_ electrochemical conversion to formic acid (HCOOH) that is in the spotlight to sustainably overcome the rising demand for chemicals within the low-carbon transition.^29^ Our results show that the sustainability level of the fossil-based chemical can be improved substantially by adequately selecting the energy source ETC. The new approach unfolds new avenues for including absolute sustainability criteria in process design.

## 2 Primary Role of Life Cycle Assessment as a Tool for Decision-Making

**Figure 1:**
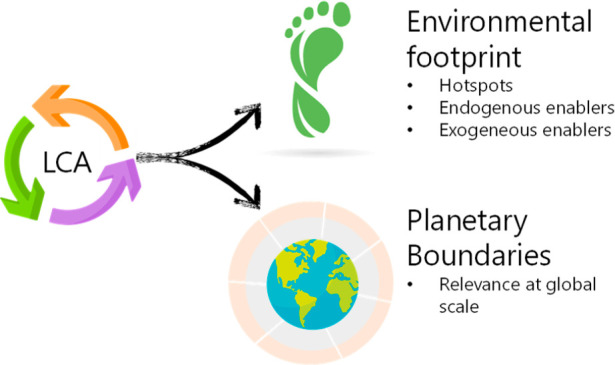
Role of life cycle assessment.

For decades, the chemical industry has put efforts into reducing chemical pollution with cleaner technologies and processes. Many efforts made to reduce some pollutants of wastes have resulted in an increased discharge at the end of the pipe, so shifting the environmental burden and impacts among environmental compartments. The LCA is a decision-making tool commonly used by designers, regulatory agencies, and organizations. According to ISO 14040, LCA can be used to assess the environmental impacts of products, processes, and services (Figure 1). LCA may also identify hotspots in which a product or process’s life cycle has the greatest reduction potential in terms of resource requirements and emissions. This is especially useful within the design stage as the environmental criteria may be included besides the traditional cost–benefit approach of designers. New approaches try to establish a link between the environmental impacts, operation, and economics of processes and technologies. The prospective application of LCA to low technology readiness level (TRL) technologies has gained momentum to enable the development of these technologies on a higher performance stage. However, limited data, uncertain functionality, scale-up issues, and uncertainties make it very challenging.^30^

LCA research applications commonly use prospective scenarios that are built, including a possible picture of future conditions at a particular point in time or describing the evolutionary pathways.^31^ Some scenarios may be based on a time horizon that is fixed in accordance with the time scale of key strategies, for example, using those decarbonization goals fixed in cornerstone scenarios to 2030 and 2050.^16^ On the other hand, the so-called “what-if” scenarios are widely used for analyzing emerging technologies, including a set of hypotheses that provide information based on low/high technology performance or varying key performance parameters to define the target performance.^32^

Defining the system boundaries is one of the first steps when carrying out an ex-ante analysis of emerging technologies. System boundaries tend to be created on attributional cradle-to-gate perspectives (from raw material extraction to industry/service gate) as the main purpose is to find hotspots and, hence, goal performances of the targeted technologies. However, the consequential viewpoint, which is commonly applied in energy systems, allows for a broad system expansion providing information that goes beyond the foreground system, tracking how environmental burdens vary in response to changes with market implications in a specific life cycle.^33−35^ Despite the complementary information that may provide the LCA consequential application evaluating decisions and cause–effect chains of more complex systems,^36^ the attributional LCA perspective is still the preferred option as a first approach to analyze the future performance of emerging technologies given the difficulty in obtaining data and the relatively low quality at low TRLs.

Another challenge when analyzing low-TRL technologies is the effect of the scale-up in the selection of the functional unit and the effect on the primary data as they may change. Two approaches are recommended to select the functional unit: (i) fix a specific function and explore a broad range of available technologies with similar functions and (ii) conduct cradle-to-gate analysis of emerging technologies with potential functions, which can be used as building blocks of future studies.^18^ Primary data, which is needed to create the life cycle inventories (LCIs) of the above-mentioned scenarios, may be obtained from experimental results, simulation data, scientific articles, patents, or even expert opinions. Furthermore, primary data obtained at time *t*_0_ should contemplate the scale effect at future time *t*_f_, so using engineering-based frameworks or scaling factors is highly recommended.^37,38^ For example, consider the reduction of the electricity consumption of large-scale devices or the increased efficiency of steam engines.

No doubt, the large number of assumptions taken during an LCA performance involves the necessity of assessing uncertainties through sensitivity analysis. Some examples found in the literature include the Monte Carlo simulation,^39,40^ which is the most common approach, the Latin hypercube approach,^41,42^ or the quasi-Monte Carlo sampling.^43^ Given the broad use of LCA results (e.g., policy makers, marketing), the communication of results under uncertainty could be critical, and they should be provided to ensure transparency and credibility to avoid biased interpretations from nonexpert stakeholders.^39^

## 3 from Environmental Footprints to Planetary Boundaries

So far, the development of emerging decarbonization technologies has been supported by single approaches, which means that target scenarios have been elucidated mainly at the environmental midpoint level or from the economic perspective. Since the introduction of the ecological footprint definition in 1992,^44^ several footprint indicators have arisen to measure a wide range of environmental burdens such as carbon footprint, water footprint, and energy footprint, among others. In this sense, the LCA framework has been traditionally based on the identification of a cause–effect chain that connects pressures to potential impacts by means of common midpoint and end-point indicators.^45^ Midpoints categories are considered to be links in the cause–effect chain of an impact category (the same environmental mechanism), whereas end-points are used to structure midpoint results by weighting or aggregation across categories reflecting damage at one of three areas of protection, which are human health, ecosystem quality, and resource scarcity.^46^

In the field of decarbonization technologies, carbon footprint and resource consumption indicators have been the preferred categories in decision-making, especially in CO_2_ conversion technologies,^17^ whereas other specific indicators, such as water footprint, have been used to evaluate green hydrogen production routes^47^ and eutrophication, land occupation, or toxicity in the field of biopolymer production.^48,49^ Midpoint environmental impact assessment provided new perspectives allowing for the identification and quantification of the benefits and targets that may boost emerging technologies to higher performance. Despite future performance scenarios being identified in the last years, full environmental sustainability remains unclear as they were not compared using those thresholds related to the planet’s capacity that were defined as planetary boundaries.^21^

Considering that the current human demand for natural resources has increased by 70% since 1970, the Earth’s natural ecosystem state is undergoing severe damage.^64^ This has led to an “ecological bottom line” that can be used to measure the sustainable development chain. The novel LCA-PBs framework^21^ could provide an approach to measure sustainability using up to 11 absolute environmental PBs that take into account the Earth’s capacity. After the combination of the selected PBs, an operating space can be defined for Earth resilience, which should not be overstepped by any of the PBs. Combining LCA-PB is a powerful methodology for decision-making when evaluating systems that can be potentially deployed at a large scale.^50^ The work reveals the potential of LCA and LCA-PB methodologies to assess the transition of the HCOOH acid market to low-carbon production. The combination of both approaches provides crucial insights into the long-term decarbonization of the EU chemical industry.

## 4 Assisting the Low-Carbon Production of Formic Acid by the LCA-PB Approach

### 4.1 Guiding Process Development with Process System Evaluation: Environmental Footprint Approach

In this section, we provide an example of how environmental footprints could be applied to guide the development of emerging technologies by giving thoughtful insights using comparable environmental metrics. The Life Cycle Impact Assessment (LCIA) method used in this section is ReCiPe 2016 v1.1 Midpoint (H), first in the category of climate change and later including freshwater consumption and land use. Ecoinvent 3.9 database^51^ and openLCA 1.11.0^52^ as software were employed. We do use the case study of producing HCOOH by CO_2_ electroreduction (CO_2_ER), an emerging CO_2_ utilization technology that is becoming more and more mature (TRL 4–5) with some demonstrations at low-scale/pilot plant level.^53,54^ The functional unit defined is to produce 1 kg of HCOOH (85% wt purity). This chemical product is conventionally produced from fossil resources, mainly using the hydrolysis of methyl formate from carbon monoxide and methanol. The cradle-to-gate carbon emissions of this route fall around 2.85–3.74 kg CO_2e_/kg.^51^ Its significant market of ∼0.71 Mton globally in 2021^55^ and promising uses as a hydrogen carrier make it an interesting chemical vector to be decarbonized.

The utilization of CO_2_ for producing HCOOH by means of an electrochemical device offers the possibility to transform a secondary source of CO_2_ (i.e., captured from industrial flue gases) by exchanging (renewable) energy, mainly in the form of electricity. The process is performed in an electrochemical reactor. On the anode side, water oxidation appears, producing oxygen (O_2_). On the cathodic side, the CO_2_ reduction takes place. This reduction would ideally only form the desired product (HCOOH in this case), but parallel reaction routes toward other compounds (methanol, ethylene, carbon monoxide, ...) and reduction of protons to form hydrogen (H_2_) also take place, reducing the net selectivity.

**Figure 2:**
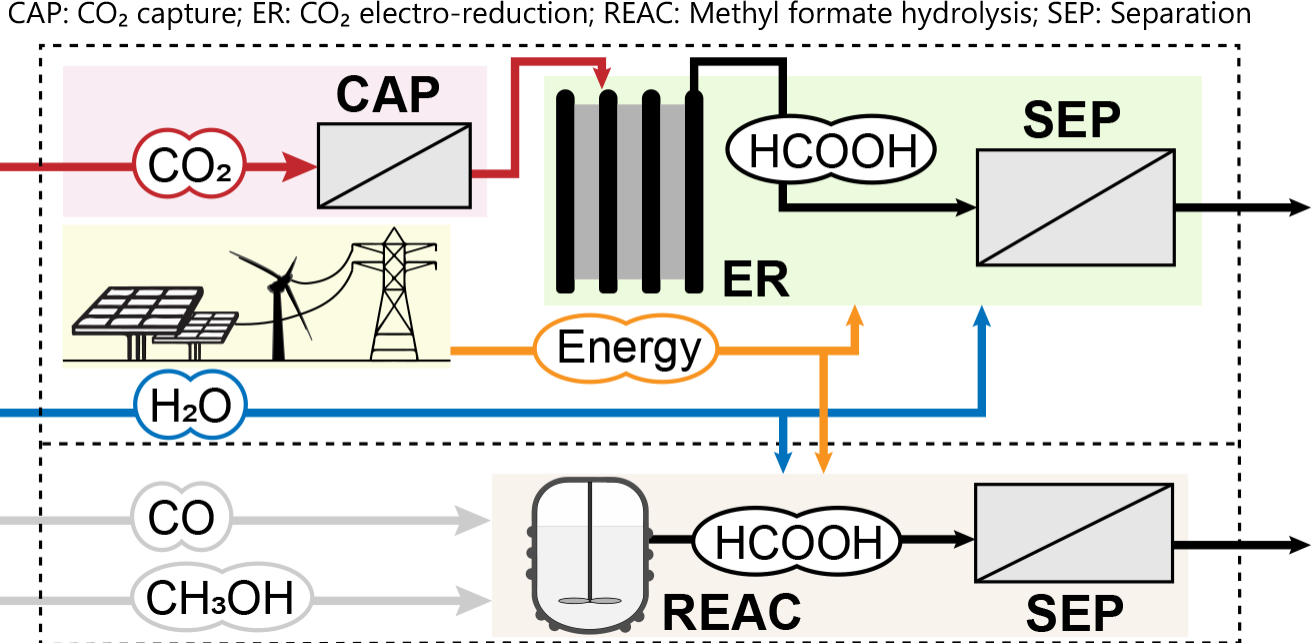
Cradle-to-gate system boundaries for CO_2_ER and fossil routes of HCOOH production. The functional unit for the case study is defined as producing 1 kg of commercial HCOOH.

Additional separation units are needed to recover unreacted CO_2_ and to purify the HCOOH in the liquid stream up to commercial purity. From a system perspective, the high energy needs through the process (electrochemical reaction, distillation of product, CO_2_ recovery) and the material requirements of the technology (electrolyzer, water, chemicals, ...) make unclear the benefits over the conventional production route. A simplified unitary process scheme is shown in Figure 2.

In this direction, environmental assessments can be used as a prospective analysis to determine hotspots in the system^10^. The constraints that affect the environmental performance of the system can be classified as (1) endogenous, when related to technology-performance variables (i.e., electrolyzer efficiency, selectivity, ...), and (2) exogeneous, when associated with external variables of the technology itself (e.g., heat and electricity source, CO_2_ source, byproduct valorization, ...). In the case of HCOOH production by CO_2_ER, a set of conditions was defined to evaluate this production route in a “baseline” scenario (based on electrochemical performance assumption from ref (56)).

**Figure 3:**
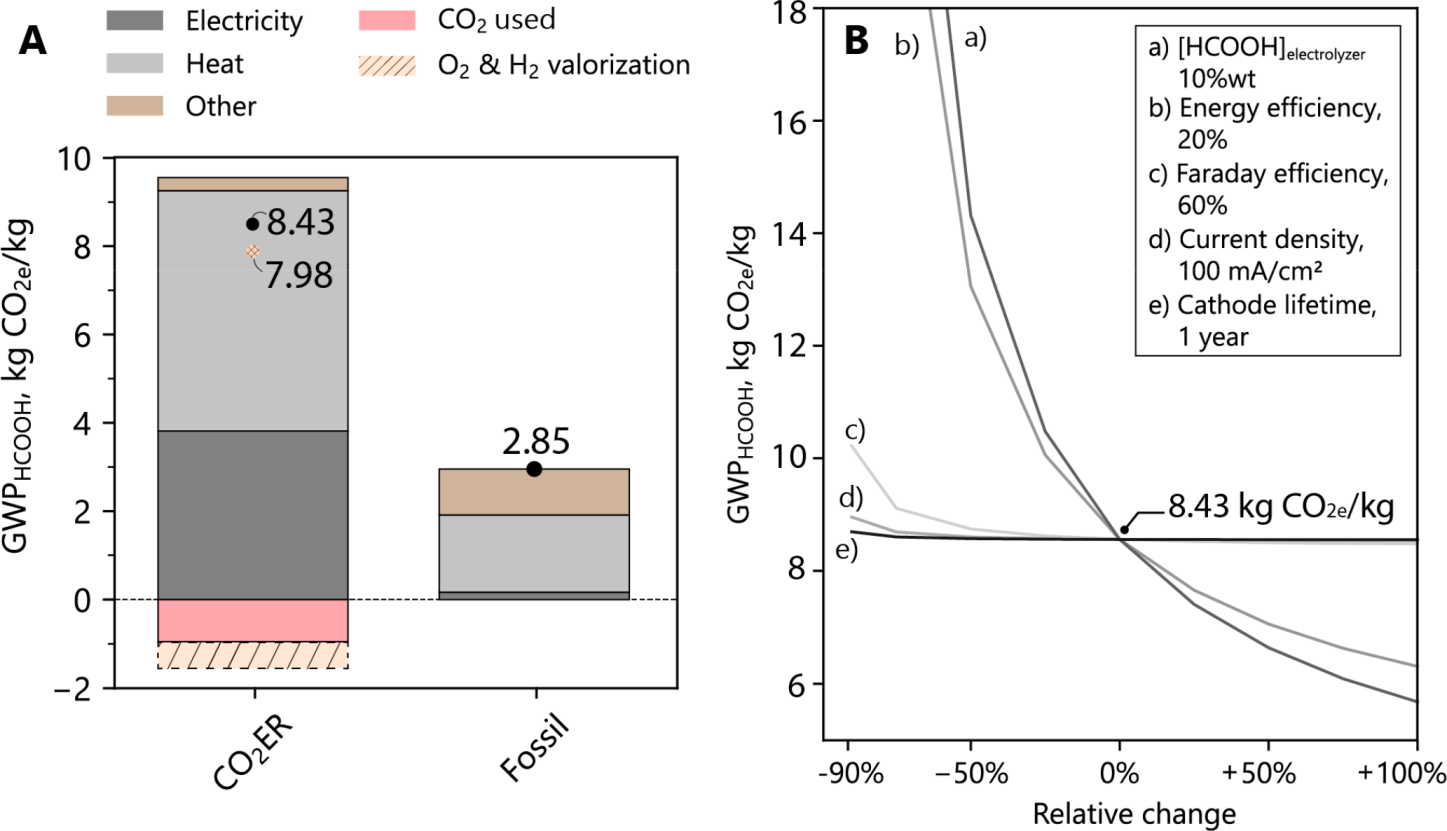
(A) Cradle-to-gate global warming potential (GWP) of HCOOH production from methyl formate hydrolysis (fossil) and CO_2_ electroreduction (CO_2_ER). The fossil route is calculated using LCI inventory from ref (51), and the CO_2_ER route uses the model from refs (17 and 57). (B) Sensitivity analysis of the GWP from the CO_2_ER route as a function of electrolyzer variables (endogenous conditions). Base values are given in the legend for each variable, which is varied individually from −90% to +100%.

Using the environmental footprint approach, the cradle-to-gate carbon emissions of the CO_2_-based route were calculated using as indicator the Global Warming Potential (GWP) and compared with the fossil-based route (Figure 3A). Additional details on the methods can be found in previously published works.^17,57^ It should be noted that the inlet CO_2_ is considered as a negative emission coming from industry, though carbon source allocation may be considered.^58^ Byproducts, when valorized, are considered as avoided-products from conventional production routes. Results showed that, under the given assumptions, the CO_2_ER route has significantly higher cradle-to-gate CO_2_ emissions, at around 9.14 kg CO_2e_/kg. These CO_2_ emissions are partially compensated by the avoided emission of the CO_2_ captured and used (0.956 kg CO_2e_/kg), as well as the potential valorization of the byproducts (0.44 kg CO_2_/kg). An individual analysis of the steps considered revealed that the major contributors in terms of CO_2_ were the electricity consumption of the electrolyzer (2.96 kg CO_2e_/kg, 31.5%) and the heat needs in the distillation (4.87 kg CO_2e_/kg, 52.2%).

Based on these results and using the process model developed for the CO_2_ER route,^16^ a sensitivity analysis of the performance variables was done to better understand the system (Figure 3B). The sensitivity chart represents the individual improvements in the GWP by changing a specific variable related to how the electrolyzer works to a certain degree. In this case, it can be clearly identified that the system’s environmental performance is endogenously conditioned by the energy efficiency (i.e., cell overpotentials) and the HCOOH concentration achieved in the ER, which later affects the distillation unit. From a carbon emission viewpoint, it can be concluded that the best improvement path is achieved by strategies related to reducing energy losses (cathode materials, cell design, change anodic reaction, ...) and testing the operation with concentrated HCOOH streams on the cathode side. Otherwise, variables such as the faradaic efficiency or the current density may not be so critical for achieving a low-carbon HCOOH production by CO_2_ER, and so research efforts should focus on enhancing first those performance variables that more restrict the system.

While the improvement in the endogenous conditions does have significant importance, exogenous ones need to be considered to define scenarios where the implementation of the CO_2_ER is truly beneficial. In this way, the environmental footprint by means of LCA can be able to assess specific scenarios attending to temporal/spatial variability, uncertainties, and decision factors that can have a significant impact on the sustainability of the process.

**Figure 4:**
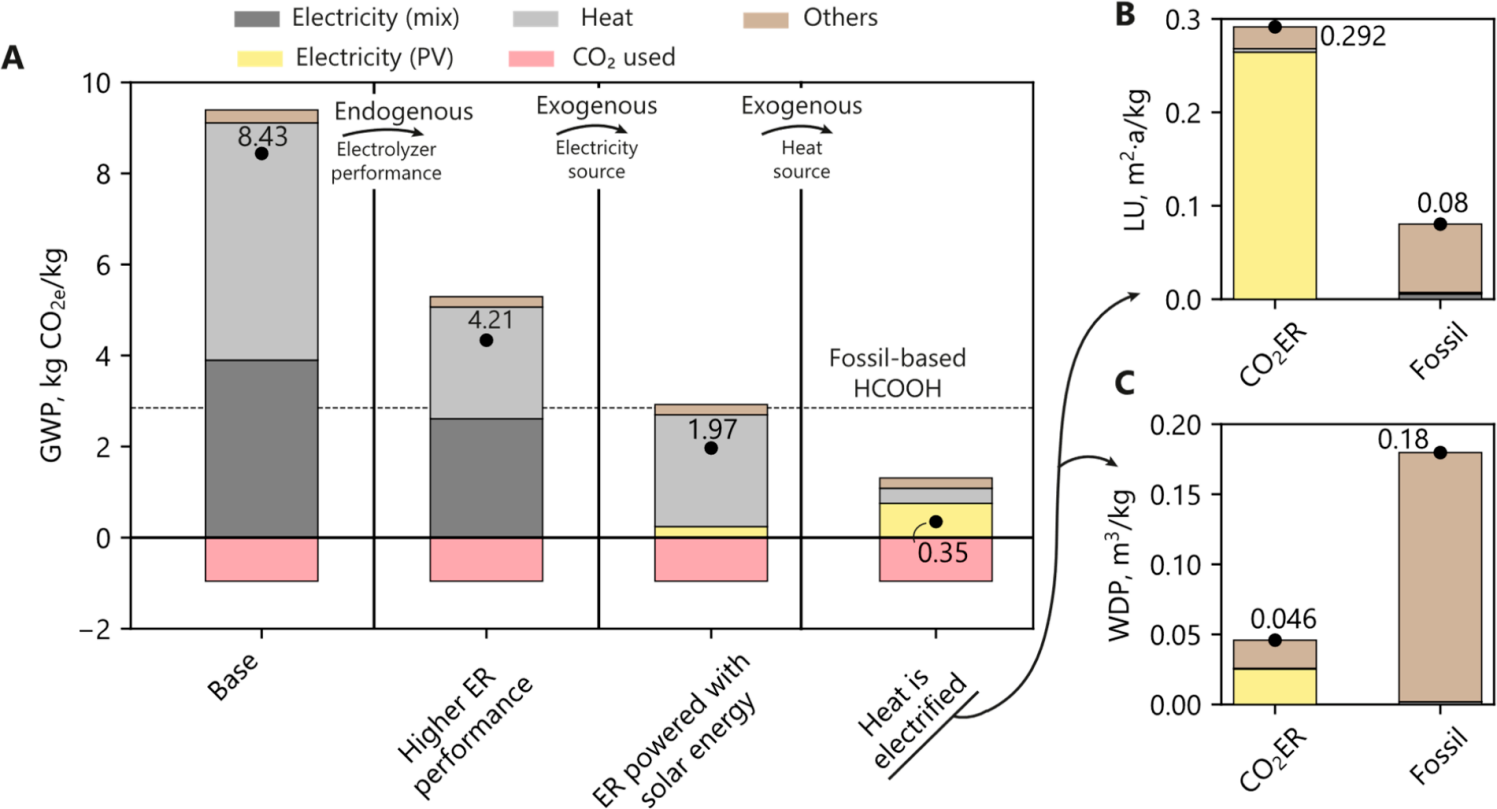
(A) Global warming potential of the CO_2_ER route under baseline and improvement scenarios. ER performance assumes increasing the energy efficiency up to 40% and HCOOH preconcentration up to 20% wt. PV energy comes from the Ecoinvent database,^51^ while heat electrification assumes an electric boiler.^60^ All steps are additive. The fossil route^51^ is shown in the dotted line (2.95 kg CO_2e_/kg,). Land use (LU) in m^2^·a (B) and water depletion potential (WDP) in m^3^/kg (C) of CO_2_ER and fossil routes. CO_2_ER uses the best-case scenario. All values are referred to the production of 1 kg of HCOOH.

Applied to the HCOOH production, several alternative scenarios are defined as variations of the “baseline” from Figure 3A and assessed together as progressive steps (Figure 4A). First, a set of improvements in endogenous conditions is evaluated according to best-performing low-scale works.^59^ This would reduce the GWP of the CO_2_ER route to 4.21 kg CO_2e_/kg, still performing worse than conventional fossil production.

Now, we do evaluate the most key exogenous conditions to seek further improvements. In the assessment the electricity and the heat source are considered. Given the inherent electricity demand of the electrochemical device, supplying a low-carbon electricity source seems critical. We do consider average Si–PV solar technology as a renewable electricity source, with carbon intensities around 35–64 kg CO_2e_/kWh.^51,62^ It could be supplied well by means of its own installed panels or by Power Purchase Agreements. Onshore wind, offshore wind, or even future electricity mixes with higher penetration of renewable energy could be the main alternatives to be explored in each specific regional situation to provide low-carbon electricity into the system. Changing the electricity source to PV solar drops the GWP of the CO_2_ER to 1.97 kg CO_2e_/kg.

Additional advances would be related to the heat as the remaining major contributor. Alternatives in this sense are electrification with electric boilers or the use of emerging heating systems using H_2_. In both cases, they should be combined with low-carbon electricity sources. Assuming an electric boiler together with PV solar energy, the GWP of CO_2_-based HCOOH would be 0.35 kg CO_2e_/kg, which from a cradle-to-gate perspective would mean reducing the CO_2_ emissions from the fossil production very significantly and having an almost carbon-neutral production.

It should be noted that the scope of the LCA assessments is highly dependent on the technological process. Regarding CO_2_ utilization technologies, the critical category for ensuring viability when comparing alternatives is climate change (carbon emissions). However, environmental sustainability can be compromised if no other indicator is considered, as adequate implementation decisions need to take into account potential trade-offs between different environmental impacts. We do calculate for the CO_2_ER route two other environmental indicators: the water consumption potential (WCP, m^3^/kg) and the land occupation potential (LOP, m^2^·a/kg). They are assessed under the best-case scenario after endogenous and exogenous improvements. Results show that the fossil route outperforms the CO_2_ER route with a significantly reduced land use (Figure 4B), while presenting an increased water use (Figure 4C). For the land use consideration, this is because of the higher electricity needs per kilogram of HCOOH and the higher land use intensity of PV solar technology compared with fossil ones. Regarding water use, the main consumption is due to the electrolyzer and distillation unit (45%), which after the considered increase in performance have a reduced water footprint when compared with the fossil route.

### 4.2 From Specific Impacts to Global Impacts: Planetary Boundaries Approach

This section briefly describes an alternative assessment approach based on the Planetary Boundaries; within this approach not only the individual footprints of a process but also its implications on a larger scale are calculated. Following the case study of HCOOH production from fossil and CO_2_ER routes, we do calculate according to updated methodology^63^ the categories related to climate change (energy imbalance and CO_2_ concentration), water use (freshwater use), and land use (land-system change). We did use the methodology proposed by Steffen et al.,^22^ using the characterization factor’s model from Ryberg et al.^63^ Among limitations, this study does not account for regional variations among processes, and global average values are used. It must be stated that the results are subject to significant uncertainty, given the low TRL level of the CO_2_ER and the lack of a worldwide consensual methodology to apply the PB assessment.

**Figure 5:**
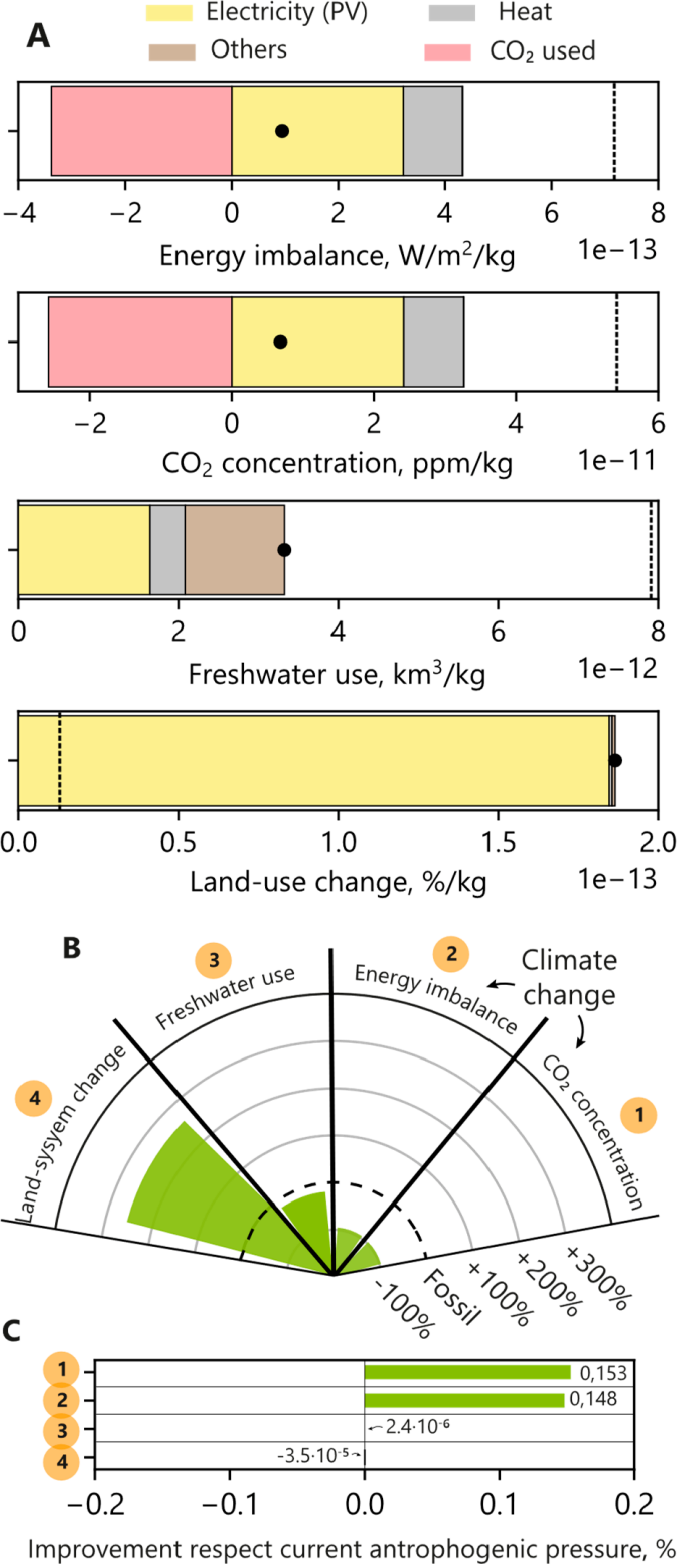
(A) Environmental performance in selected PB indicators for producing 1 kg of HCOOH in CO_2_ER route (best-case scenario). The fossil route is noted as a dotted line. (B) The ratio between the environmental burdens for the production of HCOOH by CO_2_ER and fossil routes. (C) Change in the level of transgression from anthropogenic pressures when HCOOH production changes from the fossil to the CO_2_ER route.

Using the best-case scenario for CO_2_ER from the previous section, the current anthropogenic pressure in specified categories from fossil HCOOH production and alternative CO_2_-based production is assessed (Figure 5A). These anthropogenic pressures follow similar trends as in the previous environmental footprints. However, as now the indicators are closely related to specific end-point impacts, it is possible to quantify changes in the transgression level of the planetary boundaries when switching from fossil-based to CO_2_-based production.

The environmental burdens of the fossil- and CO_2_-based HCOOH production are compared (Figure 5B) and used to calculate the benefits in the level of transgression regarding the Safe Operating Space (Figure 5C). It can be concluded that trade-offs appear between benefiting a lower-carbon economy (−91% of the pressure in CO_2_ concentration and energy imbalance), while having a similar impact on water availability (−16%) but a very relevant land use impact (+264%). While this assessment could be changed by further improvements in the technologies (alternative separation, electrolyzer materials) or by using different low-carbon electricity sources (onshore/offshore energy, nuclear power, ...), it remains clear that high-level implementation of chemical commodities as the HCOOH need proper assessment to make optimal decisions as well as truly inform the consumers of the benefits from alternative production processes.

## 5 Conclusions

This work gives an outlook on how environmental assessment tools such as the LCA can serve to guide and measure emerging decarbonization technologies and to build comparable frameworks with conventional processes. We describe the common principles of LCA and its potential uses to define and evaluate systems by means of an environmental footprint when applied to low-TRL technologies.

Then, we discuss the interest in supplementing this approach by global indicators like the Planetary Boundaries (LCA-PB) to be able to define scenarios with a higher level of connection to the end-point impacts. Finally, we provided a case study applying LCA and LCA-PB approaches to analyze the influence of both endogenous and exogeneous variables identifying that the system’s environmental performance is mainly conditioned by endogenous variables such the energy efficiency (i.e., cell overpotentials) and the HCOOH concentration achieved in the ER, which affects the distillation requirements.

On the other hand, because of the inherent electricity demand of the electrochemical device, supplying a low-carbon electricity source seems critical. A low-carbon electricity source as the PV solar energy may drop the GWP of the HCOOH produced by CO_2_ER to values lower than 2 kg CO_2_e/kg (close to the fossil-based HCOOH). The GWP of CO_2_-based HCOOH could be further decreased considering renewable heat from an electric boiler, reducing the CO_2_ emissions from the fossil production close to a carbon-neutral production (around 0.35 kg CO_2_e/kg in an assumed scenario). Despite the clear benefits in the GWP, other environmental categories should be analyzed. The results obtained showed that the fossil route outperforms the CO_2_ER route in the land use, while having a higher water use. The LCA-PB approach served to analyze the implications at large scale. After computing environmental pressures from both production systems, −91% of the pressure in CO_2_ concentration and energy imbalance was obtained, at a cost of severe increase in the land use. Switching the total production would mainly benefit to reduce the pressure on the climate change (−0.15% of the current anthropogenic pressure). The results indicated that improvements should be focus on alternative separation and electrolyzer materials as well as the evaluation of alternative low-carbon electricity sources. It remains clear that high-level implementation of chemical commodities as the HCOOH need proper assessment to make optimal decisions as well as truly inform the consumers of the benefits from alternative production processes. The tool hotspots lie in the development of technologies and definition of both technical and implementation conditions that need to be met to achieve environmental benefits. We think these tools are truly powerful when properly applied to help from the very beginning of the process to create and develop viable products and processes that lead us to a more sustainable world.

## Supporting Information Available

Process inventory for CO_2_ER and planetary boundaries assessment methodology (PDF)
