# Supplementary material for: The Relevance of Life Cycle Assessment Tools in the Development of Emerging Decarbonization Technologies
Source: JACS Au. 2023 Sep 28;3(10):2631–9. doi: 10.1021/jacsau.3c00276 (PMC10598561; doi:10.1021/jacsau.3c00276)
Supplement: Supplementary file 1 — au3c00276_si_001.pdf [file au3c00276_si_001.pdf]

SUPPORTING INFORMATION

The relevance of life cycle assessment tools in the development of emerging decarbonization technologies

Javier Fernández-González, Marta Rumayor, Antonio Domínguez-Ramos, Angel Irabien and Inmaculada Ortiz*

Departamento de Ingenierías Química y Biomolecular, Universidad de Cantabria, Av. Los Castros s/n, Santander, 39005, Spain

***Corresponding author:** Inmaculada Ortiz

University of Cantabria, Department of Chemical and Biomolecular Engineering; Av. Los Castros s/n, 39005 Santander, Spain

E-mail: inmaculada.ortiz@unican.es

Tlf: (+34) 942 201585

Table of contents

Supplementary Note 1. Process Inventory for CO_2ER	Page 2
Supplementary Note 2. Planetary boundaries assessment methodology	Page 3

Supplementary Note 1 – Process Inventory for CO_2ER

Inventory for the CO_2 electrolysis process is given (Table S1). Electrolyzer components (cathode, anode, membrane) are fully disaggregated on previous work.[1] Electrolyzer stack is based on the alkaline design from Zhao et al.[2] Balance of plant uses the inventory from Bareiß et al.[3] Capture of CO_2 is assumed to be performance with amine-based absorption using inventory from Giordano et al.[4] Material and energy needs (water, heat, electricity, minor chemicals) are calculated in the black-box model described in work of the research group. [5–7]

Table S1. Simplified inventory to produce HCOOH by the CO_2ER route.

Flow	Scenario		Unit
	Baseline	High performance	
Inputs			
Anode	2.46E-05	2.46E-05	m^2
Membrane	3.52E-05	3.52E-05	m^2
Cathode	2.46E-04	2.46E-04	m^2
Stack	1.23E-05	1.23E-05	m^2
Balance of Plant (BoP)	1.23E-05	1.23E-05	m^2
CO_2 captured	1.14	1.14	kg
Deionized water	0.68	0.68	kg
Electricity	10.3	6.4	kWh
Heat	65.15	30.70	MJ
Outputs			
HCOOH	1	1	kg
H_2	0.03	0.03	kg
O_2	0.58	0.58	kg
CO_2 to air	0.18	0.18	kg

Supplementary Note 2 – Planetary boundaries assessment methodology

The Planetary Boundaries (PB) assessment used in this works follows the basis described by Galán-Martín et al.[8] It allows the quantification of the performance of a certain system by defining a PB transgression metric, which relates the impact of the process with the control variables of the planetary boundaries. The comparison of the conventional (fossil) and alternative (emerging CO_2ER) systems in the transgression of each planetary boundary is performed taking into account their relative impact on the Safe Operating Space (SOS), helping to consider how the alternative scenarios can lead into impactful threats on the Earth limits. The updated PB framework [9] stablish the basis to include in the sustainability assessments the Earth limits. All PB have associated specific control variables, which permits to define a conjunctly SOS for the Earth system. The SOS connects the difference between the PB and the natural level, establishing a feasible space in which operate without compromising the natural Earth systems.

This work applies the characterization models proposed by Ryberg et al.,[10] to traduce the material and energy flows required in the production system into an impact on the PB's control variables. The equation can be defined as (eq. S1):

$$EB_{p,s} = \sum_{f \in F} LCI_{f,s} \cdot CF_{f,p} \qquad \text{Eq. S1}$$

where EB is the environmental burden per functional unit for each planetary boundary p in scenario s. LCI denotes the life cycle inventory for flow f in scenario s. CF indicates the characterization factor for flow f and planetary boundary p proposed by Ryberg et al.[10] The process inventory from Table S1 in fully disaggregated using the openLCA software[11] and the ecoinvent database.[12]

These impacts along the global production volume (~0.71 Mt [13]) can be computed into a transgression metric in each PB when subtracting the current fossil impact with the alternative CO_2ER route. Using the proposed SOS,[9] this global environmental burden can be quantified into a level of transgression for each PB (eq. S2)

$$LT_{p,s} = \frac{CEB_p - EB_{p,fossil} + EB_{p,CO2ER}}{SOS_p} \quad \text{Eq. S2}$$

where CEB_p in the current environmental burden for each PB b. SOS denotes the safe operating space for PB p.

References

(1) Rumayor, M.; Dominguez-Ramos, A.; Irabien, A. Environmental and Economic Assessment of the Formic Acid Electrochemical Manufacture Using Carbon Dioxide: Influence of the Electrode Lifetime. *Sustainable Production and Consumption* **2019**, *18*, 72–82. https://doi.org/10.1016/j.spc.2018.12.002.

(2) Zhao, G.; Kraglund, M. R.; Frandsen, H. L.; Wulff, A. C.; Jensen, S. H.; Chen, M.; Graves, C. R. Life Cycle Assessment of H_2O Electrolysis Technologies. *International Journal of Hydrogen Energy* **2020**, *45* (43), 23765–23781. https://doi.org/10.1016/j.ijhydene.2020.05.282.

(3) Bareiß, K.; de la Rua, C.; Möckl, M.; Hamacher, T. Life Cycle Assessment of Hydrogen from Proton Exchange Membrane Water Electrolysis in Future Energy Systems. *Applied Energy* **2019**, *237* (November 2018), 862–872. https://doi.org/10.1016/j.apenergy.2019.01.001.

(4) Giordano, L.; Roizard, D.; Favre, E. Life Cycle Assessment of Post-Combustion CO_2 Capture: A Comparison between Membrane Separation and Chemical Absorption Processes. *International Journal of Greenhouse Gas Control* **2018**, *68*, 146–163. https://doi.org/10.1016/j.ijggc.2017.11.008.

(5) Dominguez-Ramos, A.; Singh, B.; Zhang, X.; Hertwich, E. G. G.; Irabien, A. Global Warming Footprint of the Electrochemical Reduction of Carbon Dioxide to Formate. *Journal of Cleaner Production* **2015**, *104*, 148–155. https://doi.org/10.1016/j.jclepro.2013.11.046.

(6) Fernández-González, J.; Rumayor, M.; Domínguez-Ramos, A.; Irabien, Á. CO_2 Electroreduction: Sustainability Analysis of the Renewable Synthetic Natural Gas. *International Journal of Greenhouse Gas Control* **2022**, *114*, 103549. https://doi.org/10.1016/j.ijggc.2021.103549.

(7) Rumayor, M.; Dominguez-Ramos, A.; Irabien, A. Formic Acid Manufacture: Carbon Dioxide Utilization Alternatives. *Applied Sciences* **2018**, *8* (6), 914. https://doi.org/10.3390/app8060914.

(8) Galán-Martín, Á.; Tulus, V.; Díaz, I.; Pozo, C.; Pérez-Ramírez, J.; Guillén-Gosálbez, G. Sustainability Footprints of a Renewable Carbon Transition for the Petrochemical Sector within Planetary Boundaries. *One Earth* **2021**, *4* (4), 565–583. https://doi.org/10.1016/j.oneear.2021.04.001.

(9) Steffen, W.; Richardson, K.; Rockström, J.; Cornell, S. E.; Fetzer, I.; Bennett, E. M.; Biggs, R.; Carpenter, S. R.; de Vries, W.; de Wit, C. A.; Folke, C.; Gerten, D.; Heinke, J.; Mace, G. M.; Persson, L. M.; Ramanathan, V.; Reyers, B.; Sörlin, S. Planetary Boundaries: Guiding Human Development on a Changing Planet. *Science* **2015**, *347* (6223), 1259855. https://doi.org/10.1126/science.1259855.

(10) Ryberg, M. W.; Owsianiak, M.; Richardson, K.; Hauschild, M. Z. Development of a Life-Cycle Impact Assessment Methodology Linked to the Planetary Boundaries Framework. *Ecological Indicators* **2018**, *88*, 250–262. https://doi.org/10.1016/j.ecolind.2017.12.065.

(11) GreenDelta. openLCA 1.11.0, 2023. https://www.openlca.org/.

(12) Ecoinvent v3.9.1, 2023. https://ecoinvent.org/the-ecoinvent-database/data-releases/ecoinvent-3-9-1/ (accessed 2023-07-17).

(13) chemanalyst. *Formic Acid Market Size, Growth | Industry Reports To 2035*. https://www.chemanalyst.com/ (accessed 2023-05-26).
